# Automated seminal root angle measurement with corrective annotation

**DOI:** 10.1093/aobpla/plae046

**Published:** 2024-09-19

**Authors:** Abraham George Smith, Marta Malinowska, Anja Karine Ruud, Luc Janss, Lene Krusell, Jens Due Jensen, Torben Asp

**Affiliations:** Department of Computer Science, University of Copenhagen, Copenhagen 2100, Denmark; Center for Quantitative Genetics and Genomics, Aarhus University, Slagelse 4200, Denmark; Center for Quantitative Genetics and Genomics, Aarhus University, Slagelse 4200, Denmark; Center for Quantitative Genetics and Genomics, Aarhus University, Slagelse 4200, Denmark; Department of Plant Sciences, Norwegian University of Life Sciences, Ås 1433, Norway; Center for Quantitative Genetics and Genomics, Aarhus University, Slagelse 4200, Denmark; Sejet Plant Breeding, Horsens 8700, Denmark; Nordic Seed A/S, Odder 8300, Denmark; Center for Quantitative Genetics and Genomics, Aarhus University, Slagelse 4200, Denmark

**Keywords:** AI, seminal root angle, QTL, root image analysis, barley

## Abstract

Measuring seminal root angle is an important aspect of root phenotyping, yet automated methods are lacking. We introduce SeminalRootAngle, a novel open-source automated method that measures seminal root angles from images. To ensure our method is flexible and user-friendly we build on an established corrective annotation training method for image segmentation. We tested SeminalRootAngle on a heterogeneous dataset of 662 spring barley rhizobox images, which presented challenges in terms of image clarity and root obstruction. Validation of our new automated pipeline against manual measurements yielded a Pearson correlation coefficient of 0.71. We also measure inter-annotator agreement, obtaining a Pearson correlation coefficient of 0.68, indicating that our new pipeline provides similar root angle measurement accuracy to manual approaches. We use our new SeminalRootAngle tool to identify single nucleotide polymorphisms (SNPs) significantly associated with angle and length, shedding light on the genetic basis of root architecture.

## Introduction

Root architecture, encompassing root length, angles, distribution, branching pattern and overall spatial arrangement, plays a pivotal role in resource capture and plant adaptation to varying environments (Lynch 1995). The heterogeneous distribution of soil resources necessitates an efficient root system that can navigate gradients of nutrient and water availability (Lynch 1995; Roychoudhry et al. 2015). Seminal root angle has been identified as a valuable proxy for understanding the root system architecture of mature plants (Christopher et al. 2013; Richard et al. 2015).

A narrow root angle enhances water and nitrogen capture, whilst a wider angle facilitates foraging in the topsoil and improves phosphorus acquisition (Lynch 2013, 2019). The seminal roots are the initial root system, which determine the primary path for water utilization in the early stages of plant growth. Traits such as seminal root angle, represent phenotypes suitable for diverse environments. A wider root angle tends to promote a shallow rooting system, ideal for areas with frequent in-season rainfall. In contrast, a narrow root angle associated with deep rooting improves access to deep soil moisture, which is particularly beneficial during dry growth periods (Manschadi et al. 2006; Ehdaie et al. 2012).

Domestication and selective breeding, primarily through indirect selection, shaped the root architectures observed in today’s cultivated varieties (Sanguineti et al. 2006; Dorlodot et al. 2007; Fradgley et al. 2020). Investigating the genetic variation in different aspects of root architecture, including seminal root angle and root length, is key to understanding the underlying genetic factors controlling these traits. Marker-assisted selection strategies, reliant on specific quantitative trait loci (QTLs) associated with root architecture, represent a promising avenue for targeted root trait improvement.

Despite the establishment of several high-throughput methods for root phenotyping (Das et al. 2015; Yasrab et al. 2019; Seethepalli et al. 2021; Smith et al. 2022; WhinRhizo n.d.), obtaining measurements of traits such as seminal root angle remains challenging. Previous studies have relied on a manual process for root angle measurement (Hargreaves et al. 2009; Jia et al. 2019; Kitomi et al. 2020; Rufo et al. 2020; Vinarao et al. 2021), which is both time-consuming and prone to inter- and intra-annotator variation. Such manual processes have an effect on study design, limiting the scale of experiments and the frequency of imaging.

To the best of our knowledge, no prior work has addressed the need for an automated seminal root angle measurement method. To fill this gap in the root phenotyping toolbox, we developed and evaluated an automated approach to measuring seminal root angle. Our approach utilizes image segmentations created using interactive machine learning (IML).

We validated our pipeline using images of spring barley (*Hordeum vulgare*) roots from rhizoboxes, comparing automatic and manual angle measurements. To highlight the utility of our proposed method, we investigated the genetic architecture controlling root angle and length. This involved integrating root phenotypes obtained from our AI-based pipeline with genetic data by employing Bayesian variable selection, a robust analytical tool, to identify specific QTLs associated with root architecture (Janss 2010).

To promote transparency and reproducibility, we makeour image dataset freely available under a CreativeCommons license at https://zenodo.org/records/7870965#.ZEp5iXZByUk and we open-source our code and make our downloadable installer available at https://github.com/Abe404/SeminalRootAngle.

## Materials and methods

### Dataset

**Figure 1:**
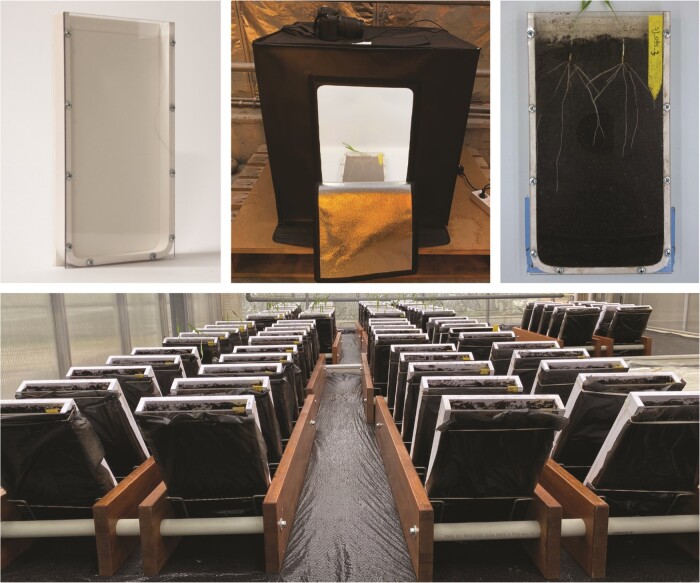
Spring barley plants growing in a rhizobox-based setup in a glasshouse. (top left) Empty rhizobox; (top centre) the light-box used in the experiment; (top right) an example of two plants in a rhizobox and (bottom) plants in the stands.

The spring barley population used in this study included 192 breeding lines and 8 commercial cultivars (Cadiz, Skyway, Laureate, Pallas, Halfdan, RGT Planet, Flair and Prospect) bred for the Nordic climate. Plants were grown in custom-made plastic rhizoboxes (Fig. 1; 20 cm × 2.5 cm × 37 cm) with a transparent plexiglass front plate. The rhizoboxes were inclined at 60° to encourage root growth along the visible side. Each box was filled with 1.8 L substrate (a mix of turf, local topsoil and sand) and supplemented with 300 mL water. Four seeds of each line were sown in each box and thinned to two seeds per box 3 days after sowing (DAS). Two seeds per line were grown in three different boxes, providing six replicates per line. Due to a limited number of rhizoboxes and the screened population size, the experiment was conducted over 4 months (August–December), and the plants were phenotyped in 15 incomplete blocks of 44 genotypes. Cultivar Skyway was included in each block as a check line. The experiment was conducted in a single greenhouse compartment with additional heating and supplemental lighting (16/8 h and 21/18° C day/night). All lines in each block were allowed to grow for 12 DAS.

At 12 DAS, roots were imaged using a Nikon D7100 camera with a Nikon 18–140 mm 1:3.5–5.6 lens in a light-photo box (Fig. 1). One high-resolution visual spectrum image (3050 × 5540 pixels) was taken of each rhizobox, and three pictures were taken of each line, resulting in 662 images in total, containing within them 1324 plants. Images were cropped and brightness corrected using a custom Python 3 script. Brightness correction employed the Yen thresholding method and intensity rescaling [see **Supporting Information**—Script 1].

### Data split

To measure the generalization of our root angle measurement method, we set aside a test set of 10 % (*n* = 66) of the images (using the random split method from the extras menu in RootPainter (Version 0.2.7)). The angle in these images was then manually measured using Fiji (Version 1.54). After excluding these 66 test images, 596 were available for segmentation model training.

### Overview of the automatic angle measurement algorithm

**Figure 2:**
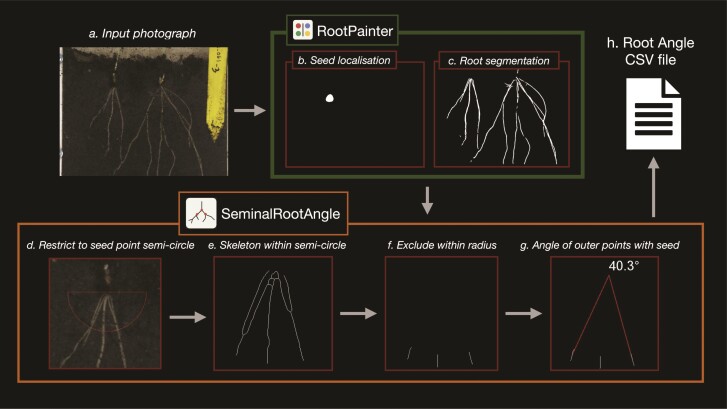
Root angle measurement pipeline. Including (A) input of a rhizobox photograph, which is segmented by two models for (B) seed localization and (C) root segmentation. The diagram illustrates how these segmentations are then used in the newly developed SeminalRootAngle software, which (D) restricts to the seed point region, (E) skeletonizes the segmented roots within this region, (F) excludes roots within a certain radius of the seed point and then (G) computes the angle between the outermost skeletonised roots, before (H) outputting to a CSV file including angles associated with all seed points found in all images.

Our new automated root angle measurement method developed and evaluated in this study consists of two stages, using the rhizobox images (Fig. 2A) a root segmentation (‘Root segmentation’ section) and seed point localization segmentation (‘Seed point localization’ section) are obtained. Then the angle measurement process (‘Angle measurement’ section) combines segmentations to measure the angle of the primary roots from the images for each seed point present in the image.

**Figure 3:**
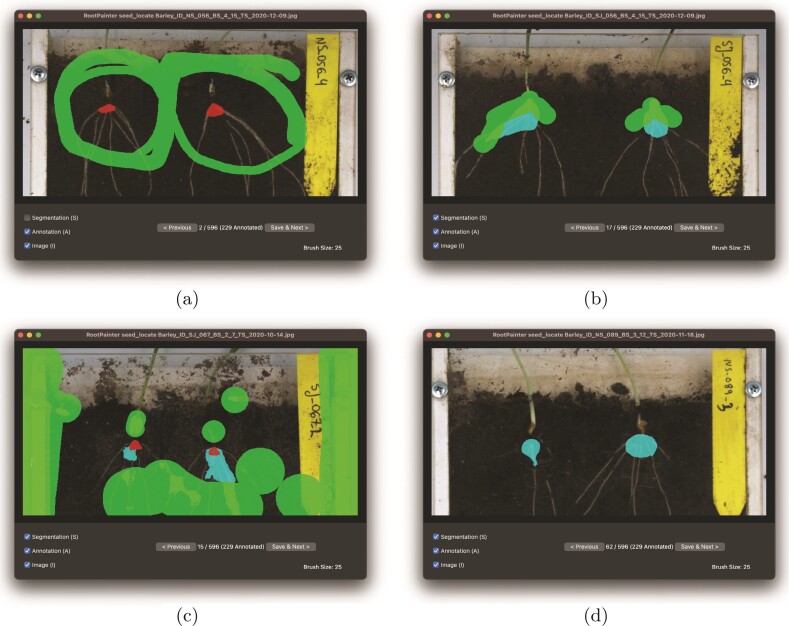
RootPainter interface showing corrective annotation at the various stages of model training for the seed localization model. (A) Seed point location annotated as a triangle and background annotated in green further away. (B and C) Model starts to predict seed points with corrective annotation used to refine seed localization segmentation model. (D) Seed point without correction.

The angle measurement algorithm, as implemented in our new SeminalRootAngle software, works by first detecting the coordinates of each seed in an image. We compute the seed as being the top central point of the segmented seed shape. To obtain the seed coordinate, we take the coordinates of each pixel belonging to each connected region in the seed segmentation. Then for connected regions over 100 pixels, we compute the *x* seed coordinate as the average of all *x* coordinates of pixels in the connected object, corresponding to the middle of the object horizontally. For the *y* coordinate, we use the pixel with the minimum *y* value, which is highest in the visible image. This centre-top location is taken into account in the way that we train the model to segment the seeds (Fig. 3A). We filter out components with less than 100 connected pixels as we assume these to be noise (small scattered false positive regions) in the segmentation and not valid seeds.

**Figure 4:**
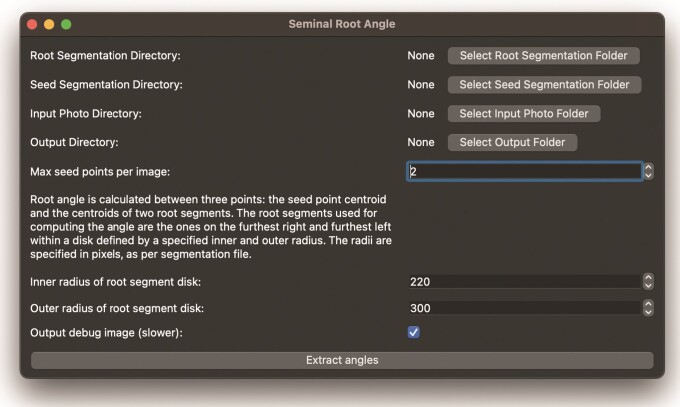
SeminalRootAngle GUI application

We then take a region around the seed point (Fig. 2D). The provided root segmentation is then skeletonized and restricted to this initial seed point region (Fig. 2E). The region close to the seed point is excluded (Fig. 2F), and the remaining outer pieces of the root skeleton are used to compute the angle (Fig. 2E). The angles associated with all seed points are output to a CSV file (Fig. 2H) including the image file name and their position in the image. In addition, to facilitate debugging, images showing each step of the processes for each seed point and a log of potential errors may be optionally output to a user-specified folder (Fig. 4).

#### Root segmentation.

The root segmentation is used for calculating both the length and, in combination with the seed segmentation (‘Seed point localization’ section), the seminal root angle. To segment the roots, we created a root segmentation model using RootPainter. To obtain this root segmentation model, we first created a training dataset with a target size of 750 pixels width and height (using the RootPainter ‘Create training dataset’ functionality), sampling three tiles from each image. The choice of 750 pixels width and height was based on the successful use of this size in Smith et al. (2022) and splitting the images up into tiles is based on results indicating that for image annotation, sampling a small region from many images is more efficient than sampling a large region from a few images (Lin et al. 2020).

We used the RootPainter corrective-annotation protocol, involving the annotation of clear regions of foreground and background in the initial images, and switched to corrective annotation (Smith et al. 2022), once the first model had been saved, which was detected by observing that the segmentation in the interface was beginning to approximate the structure of interest.

We started the network training procedure after the second image had been annotated and continued corrective annotation for a total annotation time of 3 h. Once the time limit was reached, the final annotation was saved, even if only including partial correction of the errors in the image. We then left the model to finish training on its own on the annotations produced interactively. We also took a break halfway through annotation (after 1 h and 30 min), where we let the model finish training. We used the contrast enhancement feature in RootPainter to enable darker regions of the images to be seen more clearly, as the lighting setup was suboptimal in this study. To demonstrate the effect of contrast enhancement, we include examples of before and after images in the supporting information [see **Supporting Information**—Figure S4].

We then used an ensemble (segmentation with ensembles is possible in RootPainter by selecting multiple model files when selecting the model to be used for the RootPainter ‘Segment folder’ function) of the last five output model files to segment the full root dataset.

#### Seed point localization.

Our seminal root angle measurement pipeline requires first locating the approximate seed point. To locate the seed point, we trained a seed point segmentation model in a similar way to ‘Root segmentation’ section but with the following alterations.

The seed point localization image dataset does not require fine-detailed segmentation, thus having a larger field of view to locate the seed accurately is preferred. Instead of extracting smaller tiles (subregions), we downsized the dataset. We reduced the training dataset to 25 % size (using the resize method from the RootPainter extras menu). We then cropped to the top region of the image where the seed point was contained using Fiji, as cropping to only the relevant region typically improves segmentation accuracy (Smith et al. 2023). We then trained a seed segmentation model by annotating the seed region in red (foreground) and background regions in green, whilst leaving areas close to the seed as undefined (not annotated) (Fig. 3).

Annotation was started by annotating the first clear image without regard to the model prediction (segmentation). We annotated a triangle for the seed location and annotated the background further away from the triangle as illustrated in Fig. 3A.

The seed point foreground region did not correspond to a distinct physical object in the image but a region where the roots join the stem, which may be covered with soil as shown in Fig. 3A.

After 15 images were annotated, the model started to detect the seed points. At this point, we proceeded with corrective annotation as shown in Fig. 3B and C.

After progressing through 62 images, we encountered images that did not require correction, as shown in Fig. 3D. We continued correcting mistakes in the segmentations for the entire training dataset. We spent less time training this model, with a total annotation time of 49 min and 15 s.

#### Angle measurement.

The root angle measurement function requires both the created seed point and root segmentations (Fig. 4). Both segmentation folders must be specified as input parameters. An output location for the generated CSV and a folder for debug images, to allow results to be inspected, must also be specified. Measuring root angles from the spring barley image dataset containing 662 images with debug image output disabled took 4 min and 30 s using a machine with an AMD Ryzen 9 5900X 12-Core Processor. We also output ‘debug images’ that allow inspection of the intermediary steps involved in the angle measurement process (described in ‘Overview of the automatic angle measurement algorithm’ section) and illustrated in Fig. 2. When generating these debug images, the angle measurement process takes approximately twice as long. The root photo directory is required so that debug images can be generated. We recommend generating the debug images to enable review of the angle processing steps for each image and to help understand the cause of errors.

The SeminalRootAngle GUI also provides the option to specify the maximum number of seed points per image, which is useful for rhizobox images with multiple individual plants. Root angle is calculated between three points: the seed point centroid and the centroids of two root segments. The root segments used for computing the angle are the ones on the furthest right and furthest left within a disk defined by a specified inner and outer radius. The disk radii are specified via the interface (Fig. 4) in pixels, as per the corresponding seed segmentation files.

### Evaluation of angle accuracy

To validate the automated angle measurements, we conducted manual measurements of root angles for 66 previously unseen images (corresponding to 132 seed points) in the test set. Manual angle measurements were conducted using Fiji’s dedicated ‘Angle Tool’, identified by a protractor icon. This tool allowed angle quantification by tracing lines representing the specific angles of interest within analysed images. To enable assessment of intra-annotator variability, three manual measurements were recorded for each image. To provide a comprehensive evaluation of the pipeline’s performance, automated measurements were compared to the average of the three manual measurements. We computed the Pearson correlation coefficient (*r*) and mean absolute error (MAE) to assess the correlation strength and average absolute difference between the two measurement methods. Model II regression (Ordinary Least Products) was conducted using the lmodel2 (Legendre 2018) package in R to account for measurement error in both manual and automated measurements (Ludbrook 1997; Delory et al. 2017). To assess fixed and proportional biases, confidence intervals of the regression parameters were examined. A fixed bias in the method was indicated if the 95 % confidence interval (95 % CI) for the intercept did not include zero, and a proportional bias if 95 % CI for the slope did not include one. To further evaluate the agreement between manual and automated angle measurements, a Bland–Altman analysis was performed. This involved calculating the mean difference (bias) and limits of agreement between the two methods to identify any systematic biases. To measure inter-annotator variation, 55 images (corresponding to 110 seed points) were measured by three different annotators. The inter-annotator analysis used a separate image set, distinct from the test-set used to compare manual and automated measurements. To provide insight into the efficiency of the automated angle measurement pipeline compared to manual methods, we measured the time required for conducting manual measurements.

### Genomic association study

All individuals were genotyped using either the iSelect Illumina Infinium 4k, 9k arrays or 15k SNP chip. Overlapping markers were then selected and used for the association study. To detect QTLs for root traits, we employed Bayesian Variable Selection (BVS) using a mixture prior on marker effects using the MCMC software BayZ (http://bayz.biz/ and as R-package in https://github.com/ljanss/BayzR). The BVS approach used is based on George and McCullogh (1993) and developed for QTL mapping in Heuven and Janss (Heuven et al. 2010); some details on the algorithms and implementation are given below. The fitted model for the total root length was as follows:


$$Y_{L}=1\,\mu +X\,\beta +Z\,\gamma +W\,\delta +V\,\alpha +\epsilon ,$$ (1)

where $Y_{L}$ is the vector of total root length for n plants (replicates are not specified), μ is the model mean, X is a matrix with marker genotypes with dimension n×p, where p is the number of markers. The marker data were initially coded as −1, 0 or 1, and then column-centred to mean zero. The marker effects are represented by a vector β with a dimension of p×1 and have a mixture distribution as detailed below.

Z is the design matrix capturing random effects for the block, γ is the vector of random effects parameters associated with the block and $\gamma ∼𝒩\,\left(0,I\,\sigma_{\gamma }^{2}\right)$. W is the design matrix capturing random effects for the position in the glasshouse nested within the block, δ is the vector of random effects parameters associated with the position in the glasshouse and $\delta ∼𝒩\,\left(0,I\,\sigma_{\delta }^{2}\right)$. V is the design matrix capturing random effects for growth degree nested in ID and α is the vector of random effects parameters associated with growth degree days nested in ID and $\alpha ∼𝒩\,\left(0,I\,\sigma_{\alpha }^{2}\right)$.

Model residuals are $\epsilon ∼𝒩\,\left(0,I\,\sigma_{\epsilon }^{2}\right)$. In the Bayesian setting, all model variance parameters $\sigma_{\gamma }^{2},\sigma_{\delta }^{2},\sigma_{\delta }^{2},\sigma_{\alpha }^{2}$ and $\sigma_{\epsilon }^{2}$ have prior distributions for which an (improper) unbounded uniform prior ∼U⁢(0,∞) was used. In these distributions, 𝒩⁢() denotes a Normal distribution, and I an identity matrix of appropriate size.

The seminal root angle was modelled similarly to the total root length:


$$y_{A}=1\,\mu +X\,\beta +Z\,\gamma +W\,\delta +V\,\alpha +Y\,\phi +\epsilon ,$$ (2)

where $y_{A}$ is the vector of seminal root angles for n plants. Model (2) is mostly the same as (1), but an extra effect Y⁢ϕ was added where Y is the design matrix capturing random effects for the combined effect of seed number, replicate and block and $\phi ∼𝒩\,\left(0,I\,\sigma_{\phi }^{2}\right)$ is the vector of associated random effects, and the variance $\sigma_{\phi }^{2}$ is treated the same as described above.

The BVS approach applied to marker effects β in Models (1) and (2) assumes a mixture of two normal distributions with a small variance for unselected/unimportant markers and a large variance for selected/large effect markers. An auxiliary indicator variable $\zeta_{i}\in \left\{0,1\right\}$ for every marker effect $\beta_{i}$ is used, so that $\beta_{i}∼𝒩\,\left(0,\tau_{0}^{2}\right)$ if $\zeta_{i}=0$ (small effects) and $\beta_{i}∼𝒩\,\left(0,\tau_{1}^{2}\right)$ if $\zeta_{i}=1$ (large effects). Sparse variable selection is achieved by keeping $\tau_{0}^{2}$ much smaller than $\tau_{1}^{2}$ by constraining $\tau_{0}^{2}=0.01\,\tau_{1}^{2}$, and by using $\mathrm{Pr}\left(\zeta_{i}=0\right)=\pi_{0},\mathrm{Pr}\left(\zeta_{i}=1\right)=\pi_{1}=1-\pi_{0}$, where $\pi_{0}$ is learned from the data with a prior distribution $\pi_{0}∼\text{Beta}\,\left(100,1\right)$ that implies $\pi_{0}$ to be close to 1 and most marker effects to be small.

Implementation details include joint sampling of $\beta_{i}$’s in blocks of 10 from conditional multivariate normal distributions, joint sampling of $\zeta_{i}$’s in groups of 2 after integrating out marker effects to improve mixing of the indicator variables and the use of a Metropolis–Hastings sampler to update the variance parameters $\tau_{0}^{2}$ and $\tau_{1}^{2}$ with the constraint fixed ratio.

The QTL mapping is based on using posterior means for the $\zeta_{i}$ indicator variable as a posterior probability of inclusion and a Bayes factor of the posterior and prior inclusion odds as a test statistic (see details in Heuven et al. (2010)). Computation of posterior means and standard deviations for all model parameters was performed using a single long Markov chain with a total of 250 000 iterations and a burn-in of 20 000 iterations.

## Results

### Validation of root angle

**Figure 5:**
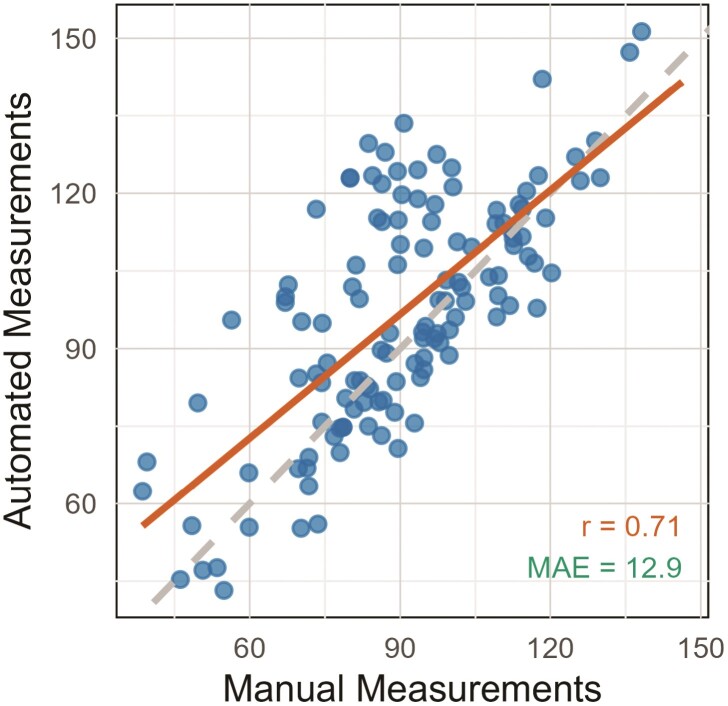
Scatter plot of manual vs. automated root angle measurements. The solid line represents the linear regression line fitted to the data. The dashed line represents a perfect 1:1 correlation. r, Pearson correlation coefficient value; MAE, mean absolute error.

The mean automated root angle was 95.58°, while the mean manual angle was 90.70°. Measurements ranged from 43.20° to 151.30°. There was a strong correlation between the manual and automated methods (*r* = 0.71) with a MAE of 12.9° (Fig. 5). Model II regression analysis indicated both fixed and proportional bias between the manual and automated measurements. The intercept (24.78, 95 % CI: 11.66–37.90) suggested a systematic overestimation by the automated method at lower values, while the slope deviating from 1 (0.80, 95 % CI: 0.66–0.94) suggested a relative underestimation for higher values. The overestimation bias was confirmed by Bland–Altman analysis, showing a mean difference of −6.59° with limits of agreement ranging from −38.86 to 25.68°. Correcting the automated measurements for these biases resulted in a higher MAE (16.61°) leading to a less accurate set of corrected measurements. Therefore, universal bias correction was not applied. Correlation coefficients between pairs of manual measurements from different annotators ranged from 0.61 to 0.80, with MAE values varying from 12.03° to 14.38° [see **Supporting Information**—Table S2]. Fixed and proportional biases were identified between each pair of annotators [see **Supporting Information**—Table S2]. Coefficients between repeated manual angle measurements from the same annotator ranged from 0.82 to 0.9 with an average MAE of 8.54 [see **Supporting Information**—Table S1].

On average, individuals performing manual measurements spent between 50 and 81 s on each image. None of the annotators did the measurements in one sitting; several breaks were necessary.

Images used in validation varied in quality, from high-quality with clearly visible roots and seeds to those with significant root obstructions and barely visible seed points.

We analysed the consistency and reliability of the correlation measurements across permutations of image order and increasing image counts [see **Supporting Information**—Figs S1 and S2]. In all cases, the standard deviation approached zero, indicating minimal variability. Correlation coefficients stabilized consistently around the 100-measurement mark (**Supporting Information**—Figs S1 and S2), with slightly more iterations needed (around 120 measurements) for the correlation between automated and manual measurements. Fluctuations in standard deviation with increasing image count and varying order underscore the importance of selecting a sufficient number of images for validating the automated pipeline against manual measurements.

### Bayesian variable selection

**Figure 6:**
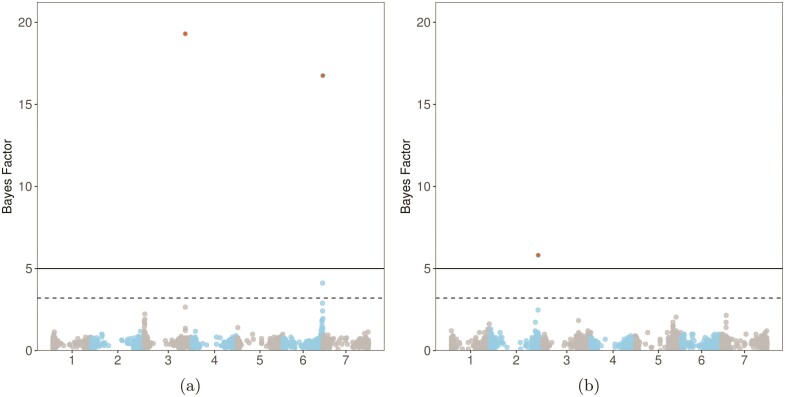
Manhattan plot of SNP for (A) the total root length and (B) seminal root angle. SNP above the line are considered significant; SNP above the dashed line are considered to be putative.

The Bayesian variable selection approach for TRL and SRA allowed us to identify significantly associated markers in the analysed population of spring barley (Fig. 6 and Table 1). Two QTLs for TRL were identified, one within an intergenic region on chromosome 3H, and the other within two genes on chromosome 6H. The QTL for SRA was located within a gene body on chromosome 2H. A significant difference was observed for both traits in lines carrying the minor allele and those with the major allele at each significant SNP [see **Supporting Information**—Fig. S3]. However, these differences were observed with small effect sizes (Cohen’s *d* ranging from 0.16 to 0.40) (Table 1)

**Table 1:** SNPs from significantly associated genomic regions for total root length (TRL) and seminal root angle (SRA). MAF, minor allele frequency; Effect, allele effect of the SNP; BF, Bayes Factor, where a value above 5 is considered ‘strong’ evidence. SNPs with BF between 3.2 and 5 are considered ‘putative’. Cohen’s d, relative effect size.

Trait	chr	pos	snpID	Effect allele	MAF	Effect	BF	Distance to closest gene	gene_id	Sequence variant	Cohen’s d
TRL	3H	594475815	SCRI_RS_198609	T	0.23	00.03	19.30	− 44302	HORVU3Hr1G081320	Intergenic variant	0.16
TRL	6H	574051819	BOPA1_1007-651	G	0.35	− 0.01	04.11	0	HORVU6Hr1G090990	Gene	0.37
TRL	6H	577494194	BOPA2_12_31283	G	0.42	− 0.03	16.75	0	HORVU6Hr1G092480	Gene	0.40
SRA	2H	700372442	SCRI_RS_184090	G	0.41	− 0.01	05.81	0	HORVU2Hr1G103570	Gene	0.26

### Computation requirements

The SeminalRootAngle GUI application is available with installers for Mac and Windows and can be run from source on Linux. The computational requirements are minimal. A computer with a dual-core CPU and at least 2 GB of RAM should be sufficient, depending on the size of the images being processed. The RootPainter software used to generate the input segmentations is computationally demanding, with the server component requiring an NVIDIA GPU with at least 6 GB of VRAM (We recommend at least 16 GB). However, it is possible to run this via a freely available colab notebook. For further details, see Smith et al. (2022).

## Discussion

We present SeminalRootAngle, a high-throughput, user-friendly tool for measuring seminal root angles from rhizobox images. By speeding up root image processing, it proves useful for genetic studies, like Genome-Wide Association Studies.

Our proposed root angle measurement method offers speed, accuracy (depending on root visibility) and is freely available as open-source software with a user-accessible GUI. The segmentation component of our pipeline was implemented using RootPainter (Smith et al. 2022), which previous studies have demonstrated to be a flexible, accessible and efficient way to train segmentation models for a variety of root datasets (Han et al. 2021, 2022; Sell et al. 2022; Bauer et al. 2022; Malinowska et al. 2022; Banet et al. 2024), including for images obtained from rhizobox experiments (Alonso-Crespo et al. 2023; Chen et al. 2022).

The landscape of root image processing software has evolved significantly, with the emergence of several widely adopted, fully automated tools such as RootPainter (Smith et al. 2022), RhizoVision Explorer (Seethepalli et al. 2020), WhinRhizo (WhinRhizo n.d.) and Digital Imaging of Root Traits (DIRT) (Das et al. 2015). Although RhizoVision Explorer provides the frequency of ‘shallow’, ‘medium’ and ‘steep’ root angles from the entire segmented root system (Seethepalli et al. 2020), measuring seminal root or root spreading angles at the seedling stage has traditionally relied on manual methods (Jia et al. 2019) or semi-automatic software applications such as RootNav (Pound et al. 2013). To the best of our knowledge, SeminalRootAngle is the first tool to fully automate the measurement of seminal root angles from rhizobox images, offering a novel solution in the root phenotyping field.

Despite the strong linear relationship indicated by the Pearson correlation coefficient of 0.71 (Fig. 5), there were occasional discrepancies between automated and manualmeasurements (Fig. 5). Model II regression analysis confirmed that while the automated method generally aligns with manual measurements, it tends to overestimate lower angle values and relatively underestimate higher ones. These discrepancies highlight the inherent challenges of measuring root angles from images due to variability in root visibility caused by obstruction from soil debris and other objects.

Root obstruction poses a challenge for both automated and manual measurements, leading to inaccuracies and intra- and inter-annotator variation. Even with repeat measurements from the same annotator, the average Pearson correlation coefficient was only 0.85 (SD = 0.05) with an MAE of 8.54° (SD = 1.07). Similarly, comparing measurements from three different annotators resulted in a Pearson correlation coefficient of 0.68 (SD = 0.10) and an MAE of 13.35° (SD = 1.20), indicating substantial inter-annotator variability likely due to the subjective nature of root anglemeasurement.

**Figure 7:**
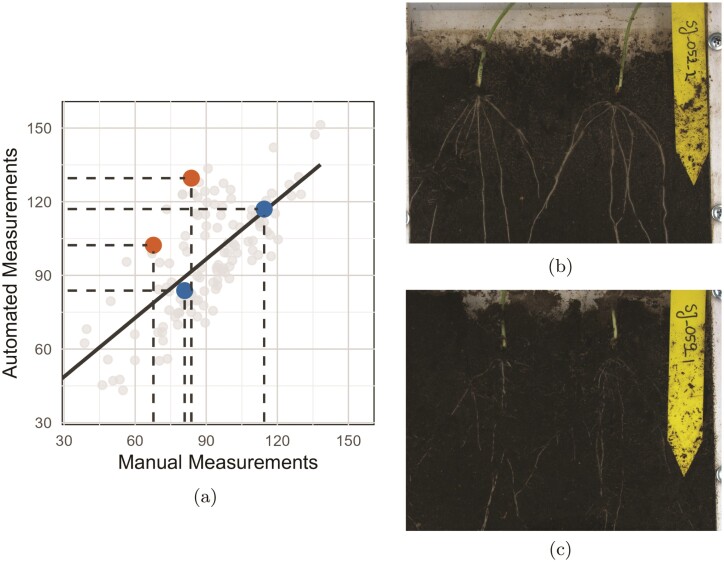
(A) Scatter plot comparing manual and automated root angle measurements, with highlighted points for two example images: (B) image with high root visibility (two highlighted blue points, overlapping with regression line) and (C) image with low root visibility (two highlighted burnt orange points, above the regression line).

The impact of root visibility on accuracy is evident when comparing images with varying quality levels. Automated measurements closely align with manual measurements in high-quality images with clearly visible roots and well-defined seed points (Fig. 7). However, significant root obstruction leads to deviations in automated measurements (Fig. 7A). This occurs because the automated method relies on locating root segmentations within a defined region around the seed point, and soil covering the root in this area can hinder detection and result in inaccuracies. As root visibility decreases, both automated and manual methods encounter challenges in identifying the roots, thus affecting accuracy in the angle measurements (Fig. 7).

Despite facing the same root visibility challenges as manual methods, SeminalRootAngle achieves accuracy comparable to human annotators. Our analysis reveals that the automated method’s error margin falls within the range of observed variability in manual measurements, demonstrating its validity and reliability even in these challenging scenarios.

SeminalRootAngle presents a substantial improvement in efficiency compared to manual methods, particularly in terms of angle measurement time. Manual processing typically requires over a minute per image and can lead to annotator fatigue, necessitating breaks and potentially extending the process over days or even weeks for datasets of average size. In contrast, our proposed pipeline significantly shortens this timeframe. For instance, in our study involving 662 images, the average time to obtain two root angles (per one image) decreased to approximately 20 s, with a further reduction to only 4–5 s when focusing solely on angle measurement. Despite the initial time investment required for pipeline training, the efficiency gains become evident, especially with larger datasets.

We identified QTLs associated with seminal root angle and total root length, aligning with the broader goal of improving crop performance through enhanced root architecture (Ren et al. 2012; Lynch 2019). We found a single QTL linked to the seminal root angle located on chromosome 2H, consistent with previous findings by Jia et al. (2019). However, our results differed from Jia et al., who identified additional QTLs on chromosomes 3H, 4H, 5H and 7H, and Robinson et al. (2018) who reported a singular QTL on chromosome 5H. In addition, we identified regions associated with total root length on chromosomes 3H and 6H. Farooqi et al. (2023) recently reported 40 QTLs for nearly 16 root architecture traits, including 2 QTLs on chromosomes 2H and 7H for total root length. Our use of advanced lines from a breeding program, compared to studies using genetically diverse materials, might explain the lower number of identified QTLs, especially considering the higher marker density and diverse germplasm employed in those studies (Jia et al. 2019). Nonetheless, our identified QTLs provide a foundation for allele stacking, offering breeders a strategic approach to optimise root architecture traits (Reynolds et al. 2020).

A limitation of our pipeline is that, in some cases, it may detect lateral roots instead of seminal roots when computing seminal root angle, leading to erroneous angle calculations. In this study, we measured plants that were 11 days old, which meant that laterals were less of a concern and we thus used a general approach to root segmentation that did not explicitly exclude lateral roots. Potential mitigations for this in future studies could be to train a segmentation model to exclude the lateral roots or to adjust the user definable inner and outer radius that determine the region of the root system to be considered for computing root angle.

Ensembles involve combining the predictions of multiple models, and have been shown to provide improvements in accuracy, even when those models are saved as part of the same training run (Huang et al. 2017). Using an ensemble slows processing time as predictions will need to be generated for each of the models in the ensemble. In this study, we used an ensemble of models to segment both the roots and the seed locations. To assess the improvements we performed an ablation analysis [see **Supporting Information**—Table S3]. The best performing model in terms of MAE and Pearson correlation was when an ensemble was used for both stages. Our results indicate that ensembles provide benefits compared to segmenting images with only a single model and may be a worthwhile approach to improving accuracy for those not bottlenecked by inference time.

For images where a clear seed point is not detected our pipeline cannot measure root angle, and for images where the seminal roots are obscured by soil the angle will likely be inaccurate. Following the approach described by Lobet et al. (2017), it may be possible to estimate seminal root angle using machine learning algorithms, such as random forest, by incorporating other available root system variables measurable from the image. The ratio between the maximum depth and maximum width of the root system has been found to have a strong and significant correlation with measures of seminal root angle (Atkinson et al. 2015), indicating, in some cases, this may serve as a proxy for images where the seminal roots are not clearly visible.

In addition to measuring seminal root angles, our method can potentially be extended to other root types, such as nodal roots. The flexibility of the IML segmentation model training method, built upon RootPainter, supports adaptation to diverse datasets. However, this requires careful training of a ‘seed localization’ model for nodal roots rather than further software development. Future work should explore this adaptability to broaden the application of our methodology.

In conclusion, SeminalRootAngle stands as a valuable tool for root phenotyping, offering a swift, accurate and user-friendly solution for measuring seminal root angles from rhizobox images.

## Supplementary Material

plae046_suppl_Supplementary_Materials

## Data Availability

We make our datasets freely available under a Creative Commons license at https://zenodo.org/records/7870965#.ZEp5iXZByUk and https://github.com/martamalinowska/BVS_for_root_traits, and we open-source our code and make our downloadable installer available at https://github.com/Abe404/SeminalRootAngle.

## Supporting Information

The following additional information is available in the online version of this article –

**Script S1.** Brightness correction of the rhizobox images.

**Table S1.** Intra-annotator agreement and mean absolute error. Pearson correlation coefficients values between each pair are below the diagonal, and mean absolute error values for each pair are above the diagonal. M1, M2 and M3 refer to three manual measurements performed by the same annotator on the test set.

**Table S2.** Inter-annotator agreement, mean absolute error and bias analysis. Pearson correlation coefficients, mean absolute error, fixed and proportional bias presence, mean difference (bias) and limits of agreement (LoA) are shown for each pair of annotators. M1, M2 and M3 refer to three manual measurements performed by 3 annotators on a set of 55 images.

**Table S3.** Comparison between Manual and the four models using a combination of a single or ensemble model for each seed localisation and root segmentation stage. The ensemble uses a weighted average of the last 5 models whilst single uses only the last model.

**Figure S1.** Variability of Pearson correlation coefficient across measurements for different permutations of image order. Points and colours indicate individual Pearson correlation coeffiecient measurements (correlations 1–10), dashed line indicate standard deviation across all measurements.

**Figure S2.** Variability of Pearson correlation coefficient across measurements for different permutations of image order between three different annotators. Points and colours indicate individual Pearson correlation coeffiecient measurements (correlations 1–10), dashed line indicate standard deviation across all measurements.

**Figure S3.** QTLs for root length and root angle traits.

**Figure S4.** Example contrast enhancement. Contrast enhancement is an option available within the RootPainter software.
