# Supplementary material for: Automated seminal root angle measurement with corrective annotation
Source: AoB Plants. 2024 Sep 19;16(5):plae046. doi: 10.1093/aobpla/plae046 (PMC11512109; doi:10.1093/aobpla/plae046)
Supplement: plae046_suppl_Supplementary_Materials [file plae046_suppl_supplementary_materials.pdf]

Supporting Information for “Automated Seminal Root Angle Measurement with Corrective Annotation"

Abraham George Smith[1,2*†], Marta Malinowska[2†], Anja Karine Ruud[2,3], Luc Janss[2], Lene Krusell[4], Jens Due Jensen[5], and Torben Asp[2]

[1]Department of Computer Science, University of Copenhagen, Copenhagen, Denmark
[2]Center for Quantitative Genetics and Genomics, Aarhus University, Slagelse, Denmark
[3]Department of Plant Sciences, Norwegian University of Life Sciences, Ås, Norway
[4]Sejet Plant Breeding, Horsens, Denmark
[5]Nordic Seed A/S, Odder, Denmark
[*]Address correspondence to: ags@di.ku.dk
[†]These authors contributed equally to this work.

1 Brightness correction script.

Listing 1: Brightness correction of the rhizobox images

```
#!/usr/bin/env python3

"""
Generic Image Brightness Correction Script

This script applies brightness correction to a set of images using the Yen
    thresholding method.

Usage:
1. Place images in the input directory.
2. Run the script to process the images and save the corrected versions in
    the output directory.

Note: Make sure to install the required dependencies before running the
    script.

```

```python
Dependencies:
- scikit-image
- imageio
- numpy
- os

"""
from skimage.filters import threshold_yen
from skimage.exposure import rescale_intensity
from skimage import io
import imageio
import os
import numpy as np

def main():
    input_directory = '/path/to/input_directory/'
    output_directory = '/path/to/output_directory/'

    # iterate through the names of contents of the folder
    for image_path in os.listdir(input_directory):

        # create the full input path and read the file
        input_path = os.path.join(input_directory, image_path)
        image_to_correct = io.imread(input_path)

        # correct brightness of the image
        yen_threshold = threshold_yen(image_to_correct)
        bright = rescale_intensity(image_to_correct, (0, yen_threshold), (0,
            255))

        # Convert the image to uint8 format
        bright_uint8 = bright.astype(np.uint8)

        # Save the corrected image to the output directory
        output_path = os.path.join(output_directory, 'bright_'+image_path)
        imageio.imsave(output_path, bright_uint8, format='JPEG', quality
            =100)

if __name__ == '__main__':
    main()
```

1.1 Intra-annotator agreement

	M1	M2	M3
M1	1.00	9.16	9.15
M2	0.82	1.00	7.31
M3	0.82	0.90	1.00

Table 1: Intra-annotator agreement and mean absolute error. Pearson correlation coefficients values between each pair are below the diagonal, and Mean Absolute Error values for each pair are above the diagonal. M1, M2, and M3 refer to three manual measurements performed by the same annotator on the test set.

1.2 Intra-annotator Correlation Variation

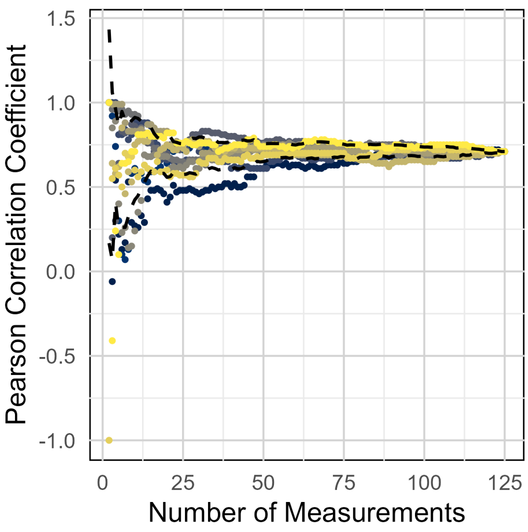

Figure 1: Variability of Pearson correlation coefficient across measurements for different permutations of image order. Points and colours indicate individual Pearson correlation coeffiecient measurements (correlations 1 to 10), Dashed line indicate standard deviation across all measurements.

1.3 Inter-annotator Correlation Variation

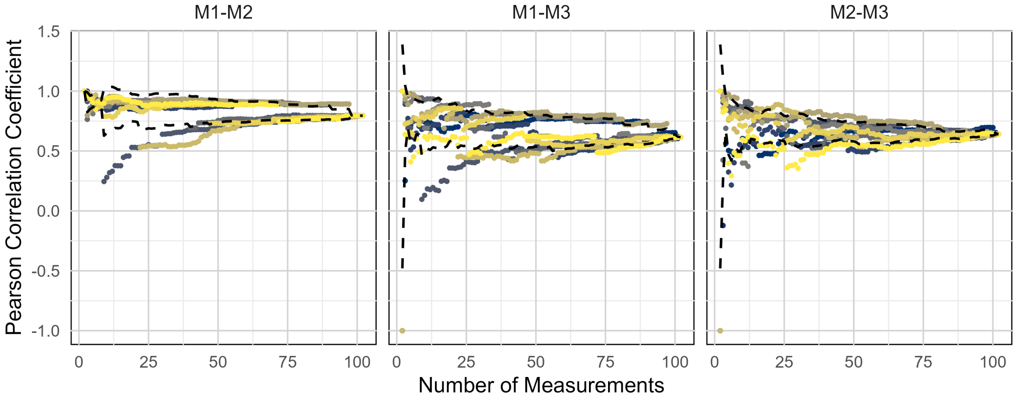

Figure 2: Variability of Pearson correlation coefficient across measurements for different permutations of image order between three different annotators. Points and colours indicate individual Pearson correlation coeffiecient measurements (correlations 1 to 10), Dashed line indicate standard deviation across all measurements.

1.4 Inter-annotator Correlation MAE

Annotators	Pearson Correlation	MAE	Fixed Bias	Proportional Bias	Mean Difference (Bias)	LoA Lower	LoA Upper
M1 vs M2	0.82	9.16	Yes	Yes	-3.48	-28.86	21.91
M1 vs M3	0.82	9.15	Yes	Yes	-0.73	-25.12	23.67
M2 vs M3	0.90	7.31	Yes	Yes	2.75	-16.40	21.91

Table 2: Inter-annotator agreement, mean absolute error, and bias analysis. Pearson correlation coefficients, Mean Absolute Error, fixed and proportional bias presence, mean difference (bias), and limits of agreement (LoA) are shown for each pair of annotators. M1, M2, and M3 refer to three manual measurements performed by three annotators on a set of 55 images.

1.5 Root length and root angle QTLs

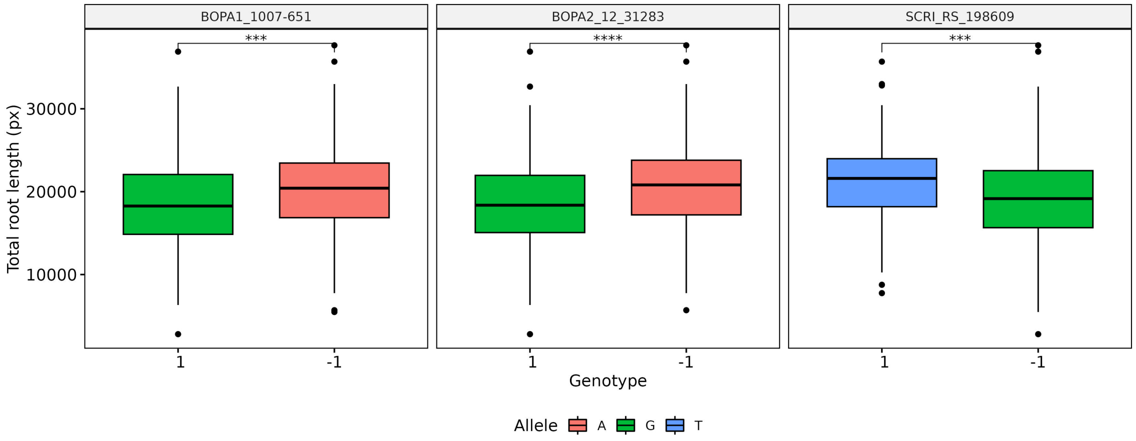

(a)

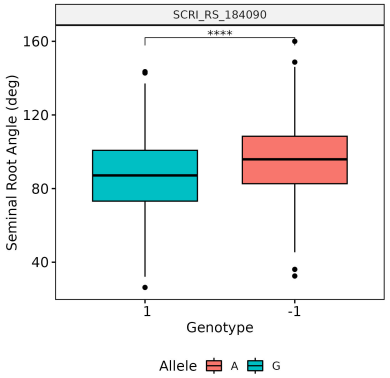

(b)

Figure 3: QTLs for root length and root angle traits.

1.6 RootPainter Contrast Enhancement

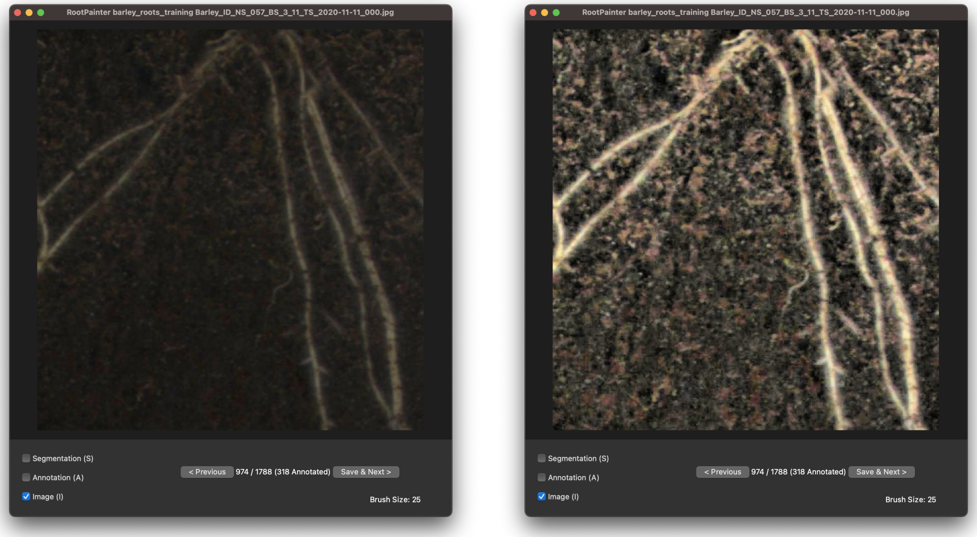

(a) Without contrast enhancement

(b) With contrast enhancement

Figure 4: Example contrast enhancement. Contrast enhancement is an option available within the RootPainter software.

1.7 Ensemble Ablation Analysis

Seed	Root	Pearson Correlation	MAE	Fixed Bias	Proportional Bias	Mean Difference (Bias)	LoA Lower	LoA Upper
Ensemble	Ensemble	0.71	12.85	Yes	Yes	-6.48	-38.92	25.95
Ensemble	Single	0.61	14.62	Yes	Yes	-4.85	-41.85	32.14
Single	Single	0.61	14.68	Yes	Yes	-5.31	-42.46	31.84
Single	Ensemble	0.69	13.52	Yes	Yes	-6.97	-40.98	27.04

Table 3: Comparison between Manual and the four models using a combination of a single or ensemble model for each seed localisation and root segmentation stage. The ensemble uses a weighted average of the last 5 models whilst single uses only the last model.
